# The Role of Choline, Soy Isoflavones, and Probiotics as Adjuvant Treatments in the Prevention and Management of NAFLD in Postmenopausal Women

**DOI:** 10.3390/nu15122670

**Published:** 2023-06-08

**Authors:** Johanna K. DiStefano

**Affiliations:** Diabetes and Metabolic Disease Research Unit, Translational Genomics Research Institute, Phoenix, AZ 85004, USA; jdistefano@tgen.org; Tel.:+1-602-343-8814

**Keywords:** nonalcoholic fatty liver disease, heterogeneity, women, menopause, estrogen, nutrition, choline, soy isoflavones, probiotics

## Abstract

Nonalcoholic fatty liver disease (NAFLD) is a prevalent condition among postmenopausal women that can lead to severe liver dysfunction and increased mortality. In recent years, research has focused on identifying potential lifestyle dietary interventions that may prevent or treat NAFLD in this population. Due to the complex and multifactorial nature of NAFLD in postmenopausal women, the disease can present as different subtypes, with varying levels of clinical presentation and variable treatment responses. By recognizing the significant heterogeneity of NAFLD in postmenopausal women, it may be possible to identify specific subsets of individuals who may benefit from targeted nutritional interventions. The purpose of this review was to examine the current evidence supporting the role of three specific nutritional factors—choline, soy isoflavones, and probiotics—as potential nutritional adjuvants in the prevention and treatment of NAFLD in postmenopausal women. There is promising evidence supporting the potential benefits of these nutritional factors for NAFLD prevention and treatment, particularly in postmenopausal women, and further research is warranted to confirm their effectiveness in alleviating hepatic steatosis in this population.

## 1. Introduction

NAFLD is a chronic condition that develops due to an excessive accumulation of fat in the liver. It is a heterogeneous disorder that encompasses a range of liver diseases with varying degrees of severity, clinical characteristics, and outcomes. This spectrum includes isolated steatosis, which is defined as the accumulation of lipids in 5% or more of hepatocytes, as well as nonalcoholic steatohepatitis (NASH), which is characterized by liver inflammation and hepatocellular injury. In some individuals, NASH can progress to liver fibrosis, cirrhosis, and hepatocellular carcinoma [1]. Histological features of NAFLD also vary, including degrees of steatosis, inflammation, ballooning, and fibrosis. Predicting the disease trajectory in individual patients is a significant clinical challenge due to these variations.

The heterogeneity of NAFLD is multifactorial, stemming from a complex interplay of genetic and environmental factors, underlying pathophysiological mechanisms, and coexisting medical conditions, especially obesity, insulin resistance, and metabolic syndrome [2,3,4,5]. Indeed, the term “MAFLD” (metabolic dysfunction-associated fatty liver disease) has recently been proposed to more accurately capture its strong association with metabolic disorders such as obesity, insulin resistance, type 2 diabetes, dyslipidemia, and hypertension [6]. MAFLD encompasses a broader spectrum of liver diseases, including NAFLD, and comprises a wider range of affected individuals, including those with lean body mass and of a normal weight. Similar to NAFLD, MAFLD can progress from simple steatosis to severe liver disease, including hepatocellular carcinoma. Such heterogeneity has significant clinical implications that can affect the diagnosis, prognosis, and treatment of NAFLD [7]. For instance, some patients may rapidly progress to advanced stages of the disease, while others may remain stable or even improve over time [8]. Therefore, a personalized approach to NAFLD diagnosis and management is essential, one which considers the various subtypes and stages of the disease and individual risk factors and characteristics of each patient.

The prevalence of NAFLD is on the rise globally, with up to one-third of adults in Western countries affected, and even higher rates among individuals with obesity, type 2 diabetes, and metabolic syndrome [5]. Following menopause, women are at an increased risk of developing both NAFLD and advanced NASH fibrosis, with a prevalence that is comparable to, or even higher, than that in men of the same age, with rates between 15–62%, depending on geographical location, ethnicity, and method of diagnosis [9,10,11]. In addition, postmenopausal women have higher rates of advanced fibrosis compared to men (36.1% vs. 17.7%) [12]. While NAFLD is generally more prevalent in men, there has been a significant increase in its prevalence among women over the past decade, from 18.5% in 1988–1994 to 21.3% in 1999–2006 and to 24.9% in 2007–2014 [13]. Women are also experiencing a steeper annual rate increase [14] and higher mortality rate compared to men [15]. Notably, NASH has emerged as the leading reason for liver transplantation in women [16].

Because NAFLD is closely linked with obesity and insulin resistance [17], lifestyle modifications are the most effective approach to treating the condition. Specifically, reducing caloric intake, limiting consumption of simple sugars and saturated fats, and increasing physical activity levels to promote weight loss and improve metabolic parameters are recommended as the first-line therapy for NAFLD patients [18,19]. Controlling underlying metabolic conditions such as diabetes, hypertension, and hypercholesterolemia can further decrease the risk of NAFLD. Modest weight loss, representing only 5–10% of the total body weight, has been shown to reduce liver fat and improve liver function [20,21], even in individuals with a normal body weight [22,23].

While it may seem reasonable to expect that lifestyle modifications resulting in weight loss and improved metabolic parameters in postmenopausal women would lead to reductions in liver fat content, there are limited studies that investigate the role of diet in NAFLD. A recent randomized dietary intervention trial discovered that both paleolithic (high intake of vegetables, fruit, nuts, eggs, fish, and lean meat, while excluding refined sugar, salt, dairy products, and grains) and conventional low-fat diets (high intake of fruit, vegetables, whole grains, and fish, with low-fat meat and dairy products) resulted in a ~50% reduction of liver fat in healthy postmenopausal women with obesity [24]. In this study, it was observed that the reduction of liver fat in the low-fat diet group was linked to weight loss, while the paleolithic diet group exhibited improvements in liver fat due to a greater proportion of mono- and polyunsaturated fatty acids. These findings suggest that different dietary patterns may provide benefit to the liver through distinct mechanisms.

In a post hoc analysis of 4162 postmenopausal women from the UK Women’s Cohort Study, higher adherence to the Mediterranean diet, which is the recommended dietary pattern for all NAFLD patients [25], was associated with a smaller increase in waist circumference and a reduced risk of abdominal obesity in postmenopausal women with overweight [26]. A cross-sectional analysis of 2000 women revealed that greater adherence to the Mediterranean diet was linked with a reduced risk of metabolically unhealthy obesity in postmenopausal women; this association was not observed in premenopausal women [27]. However, a different study found that adherence to this diet had a favorable impact on hepatic steatosis in both postmenopausal and premenopausal women, albeit with a stronger effect observed in the latter [28].

**Figure 1:**
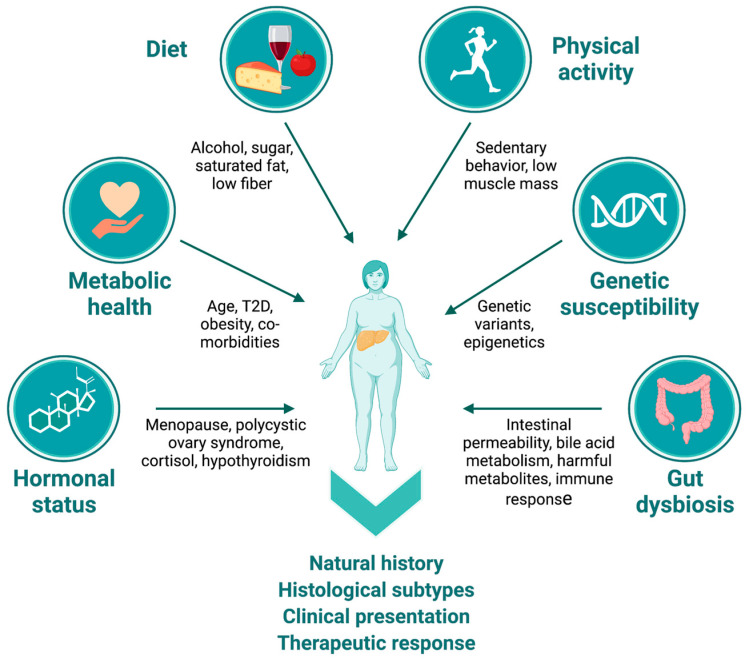
NAFLD heterogeneity in postmenopausal women. Multiple etiological factors contribute to heterogeneity in natural history, histological subtypes, clinical presentation, and response to therapeutic interventions in postmenopausal women. Factors such as hormonal status, age, metabolic health and presence of comorbidities, quality of habitual diet, alcohol consumption patterns, levels of physical activity, genetic factors/ethnicity, and presence of gut dysbiosis through diverse mechanisms often interact dynamically to affect the clinical presentation of NAFLD in this population. (Figure created with BioRender.com, accessed on 31 May 2023).

There are several factors that contribute to the complex heterogeneity underlying NAFLD that are particularly relevant in postmenopausal women. These include hormonal status, age, lifestyle factors, genetic factors, and gut dysbiosis (Figure 1). Hormones, especially sex hormones, have been shown to influence the development and progression of NAFLD, with the decline in estrogen levels after menopause contributing significantly to disease risk [29]. Increasing age is also an important factor that can affect the natural history of NAFLD in this population, as older individuals are more likely to have comorbidities that can exacerbate the disease [30,31]. Moreover, age-related changes in metabolism and hormone levels can also contribute to the progression of NAFLD [32]. Other hormonal imbalances, such as those observed in polycystic ovary syndrome (PCOS), are known to increase the risk of NAFLD [33]. Lifestyle factors such as diet, exercise, and alcohol consumption can interact dynamically with other factors to affect the development and natural history of NAFLD in all individuals, including postmenopausal women [34]. Metabolic health is a critical factor in the development and progression of NAFLD, with comorbidities such as insulin resistance, dyslipidemia, and metabolic syndrome increasing the risk of developing and progressing to NASH [35].

NAFLD is a moderately heritable condition [36], and several common genetic variants have been consistently associated with risk of NAFLD across diverse populations [37]. Genetic factors can also influence the presentation of NAFLD in postmenopausal women. For instance, the rs738409 variant in the patatin-like phospholipase domain-containing protein 3 gene (*PNPLA3*) has been found to significantly modulate the relationship between dietary intake and fibrosis severity in a dose-dependent, genotype-specific manner. Specifically, carriers of the variant allele were more affected by dietary factors such as carbohydrate, *n*-3 polyunsaturated fatty acids, isoflavones, methionine, and choline [38]. Likewise, variants in the phosphatidylethanolamine N-methyltransferase gene (*PEMT*) can worsen the effects of insufficient choline intake on NAFLD risk in postmenopausal women [39]. Additionally, epigenetic modifications contribute to heterogeneity in the manifestation of NAFLD. For example, Sokolowska et al. [40] recently found that changes in the blood methylomes of NAFLD patients in response to a Mediterranean diet were correlated with improvements in hepatic steatosis and could stratify liver biopsies by fibrosis grade.

Gut dysbiosis, or alterations in the gut microbiota, is another factor known to contribute to the development and progression of NAFLD. Dysbiosis can lead to an increase in gut-derived endotoxins, triggering inflammation in the liver and contributing to the disease [41].

Due to the complex and multifactorial nature of NAFLD in postmenopausal women, the disease can present as different subtypes, with varying levels of clinical presentation, diverse pathological mechanisms, and treatment responses. By recognizing the significant heterogeneity of NAFLD in postmenopausal women, we might identify specific subsets of individuals who may benefit from targeted nutritional interventions. The objectives of this review were to explore the increased risk of NAFLD in postmenopausal women and examine the current evidence supporting the role of three specific nutritional factors—choline, soy isoflavones, and probiotics—as potential nutritional adjuvants in the prevention and treatment of NAFLD.

## 2. NAFLD in Postmenopausal Women

### 2.1. Why Does NAFLD Risk Increase after Menopause?

The prevalence of NAFLD increases significantly in women following menopause [30,42,43,44,45,46,47], with postmenopausal women having ~2–3 times higher rates of advanced fibrosis compared to premenopausal women [11,12]. Even in women without obesity, the risk of advanced fibrosis is substantially higher compared to premenopausal women, even after accounting for factors such as disease severity, body mass index (BMI), impaired glucose tolerance or diabetes, and hypertension [48]. Notably, in postmenopausal women, a larger waist circumference is associated with a higher risk of advanced fibrosis, while in men and premenopausal women, extremity, but not abdominal, size, is linked to advanced fibrosis [48]. Central adiposity, weight gain, and metabolic syndrome represent major risk factors for NAFLD in postmenopausal women [30,43]. In addition, the loss of muscle mass, i.e., sarcopenia, can have implications for NAFLD in postmenopausal women [49]. Sarcopenia is associated with metabolic abnormalities, including insulin resistance, dyslipidemia, and obesity, which are all risk factors for the development and progression of NAFLD [50,51].

The natural history of NAFLD in postmenopausal women remains unclear; however, menopause-associated changes in the hormonal milieu contribute to the development and progression of NAFLD. Estrogen is known to have a protective effect on the liver, corresponding to improved liver fat fraction, reduced inflammation and oxidative stress, and increased insulin sensitivity [10,52]. The decline in estrogen levels after menopause has been associated with an increase in visceral adiposity, insulin resistance, and inflammation, all of which are key risk factors for NAFLD [52,53,54]. An observational longitudinal study by Lovejoy et al. [55] demonstrated that while middle-aged women accrue subcutaneous adipose tissue with age, menopause is specifically associated with an increase in total body fat and visceral adipose tissue. Menopause onset was also observed to correspond with decreased energy expenditure and reduced fat oxidation [55]. Additionally, the metabolic changes that occur after menopause, such as an increase in total body fat, a shift towards central adiposity, and a decrease in lean body mass, exacerbate NAFLD risk in this population. Furthermore, postmenopausal women are more likely to have other risk factors for NAFLD, such as hypertension, dyslipidemia, and type 2 diabetes [56,57].

### 2.2. Strategies for Risk Reduction of NAFLD in Postmenopausal Women

Various strategies seek to reduce the risk of NAFLD in postmenopausal women by targeting underlying metabolic risk factors [58]. As noted, the major strategies to improve liver function and reduce liver fat accumulation, inflammation, and fibrosis are weight loss [4] and exercise [59,60]. However, lifestyle interventions may differ by sex. For instance, a study by Vilar-Gomez et al. [20] found that while weight loss improved NASH histology in both men and women, women had a lower probability of improvement or resolution of NASH compared to men, indicating that women may need to achieve greater weight loss to realize comparable hepatic benefit.

In addition to weight loss, menopause hormone therapy (MHT) may offer unique treatment targets for women with NAFLD due to their distinct hormonal profiles. Estrogen modulation, in particular, has been considered a potential treatment modality for NAFLD in women, supported by data demonstrating the benefits of estrogen on metabolic health and liver fibrosis. A randomized controlled trial (RCT) of MHT in women with type 2 diabetes and presumed NAFLD showed improvement in elevated liver enzymes among MHT users, suggesting potential protective effects of exogenous estrogen on hepatic inflammation [61]. In a large cross-sectional study involving 251 postmenopausal women, it was found that those taking MHT had a lower prevalence of NAFLD (14 out of 53; 26.4%) compared to those who were not taking MHT (79 out of 198; 39.9%) [62]. Additionally, levels of gamma-glutamyl transpeptidase (GGT), alanine transaminase (ALT), ferritin, and insulin resistance were higher in NAFLD patients who were not taking MHT, while women who were not taking MHT, regardless of their NAFLD status, had higher rates of overweight, obesity, and insulin resistance compared to those who used MHT.

A meta-analysis of over 100 randomized trials in postmenopausal women found that the use of estrogen supplementation, with or without progesterone, can increase lean body mass and reduce abdominal fat, improve insulin resistance, and decrease blood pressure [63]. These findings suggest that estrogen supplementation may reduce the metabolically unhealthy characteristics associated with menopause and support the role of estrogen deficiency in the accrual of excess weight in postmenopausal women. The meta-analysis also showed that MHT reduces abdominal obesity, insulin resistance, new-onset diabetes, lipids, blood pressure, adhesion molecules, and procoagulant factors in women without diabetes, also improving insulin resistance and fasting glucose in women with diabetes.

Pharmacological interventions, including insulin sensitizers such as metformin and thiazolidinediones, and lipid-lowering agents such as statins and fibrates, have also been studied for the treatment of NAFLD. These medications have shown promise in improving metabolic parameters and reducing liver fat accumulation, inflammation, and fibrosis. However, their efficacy and safety in treating NAFLD, particularly in postmenopausal women, are still being evaluated. Managing co-morbidities such as hypertension, dyslipidemia, and type 2 diabetes with pharmacological interventions is also important in reducing the risk of NAFLD [58]. Tight control of glycemia, blood pressure, and lipid levels with appropriate therapies can help improve metabolic parameters and decrease the risk of developing NAFLD.

Bariatric surgery is known to improve metabolic parameters and decrease the risk of NAFLD in individuals with severe obesity, which is largely attributed to the substantial weight loss, enhanced insulin sensitivity, and reduced inflammation following surgery [64].

In summary, strategies for reducing the risk of NAFLD in postmenopausal women include lifestyle modifications; use of MHT; pharmacological interventions, including management of co-morbidities; and bariatric surgery in women with obesity. These strategies should be individualized based on the specific metabolic risk factors present in each patient.

## 3. Nutritional Factors That May Benefit Postmenopausal Women with NAFLD

While there are no specific dietary factors associated with NAFLD that are unique to postmenopausal women, the effects of some of these factors could yield greater impact on hepatic fat accumulation under conditions of estrogen deficiency. Among these dietary factors, the best studied include a high fat diet, particularly one that is high in saturated and trans fats; excessive consumption of sugar and refined carbohydrates; inadequate consumption of fiber; and mild–moderate alcohol consumption, all of which can affect the development and progression of NAFLD [65].

There is also growing evidence that specific nutritional factors may help to prevent or treat NAFLD. For example, supplementation with vitamins C, D, and E may exert beneficial effects on liver health or related metabolic features, primarily through their antioxidant and anti-inflammatory properties [66,67,68,69]. Coffee consumption is inversely associated with a number of liver-related conditions, including slower progression of fibrosis, lower transaminase levels, and decreased liver-related mortality, and some of these positive effects may be due to caffeine [70,71]. Caffeine has also been shown to improve features of metabolic syndrome, including hepatic injury in high carbohydrate, high-fat-diet-fed rats [72]; increase energy expenditure [73]; and reduce total body, trunk, and visceral fat [74] or fat mass [75]. However, these vitamins and dietary factors are not specific for the treatment of NAFLD in postmenopausal women.

Emerging evidence suggests that three dietary factors, namely choline, soy isoflavones, and probiotics, may hold promise in providing potential benefits for specific subsets of postmenopausal women with NAFLD. The importance of these nutritional factors in women with estrogen deficiency and the role they play in improving liver health are discussed in the following sections.

### 3.1. Choline

Choline is an essential nutrient that plays a critical role in liver function and is a key component of phosphatidylcholine, a major phospholipid found in cell membranes [76]. Dietary choline deficiency, even for a short duration, causes significant liver dysfunction, including hepatic steatosis, in healthy men and women [39,77,78] and laboratory mice [79]. The Adequate Intake (AI) for choline is 550 mg/day for men and 425 mg/day for women [80,81,82]. However, dietary choline requirements vary among individuals, with some requiring much greater amounts of choline to avoid the development of metabolic dysfunction [77]. There is also evidence indicating that many individuals do not regularly meet the AI recommendations for choline [80,81,82].

Phosphatidylethanolamine N-methyltransferase (PEMT) catalyzes the conversion of phosphatidylethanolamine to phosphatidylcholine [83]. Formation of very low-density lipoprotein (VLDL), which conveys triacylglycerols from the liver to the circulation, requires phosphatidylcholine. Low levels of phosphatidylcholine result in insufficient VLDL production, leading to fat accumulation in hepatocytes [83,84,85]. In mice with reduced expression of the *PEMT* gene (Pemt^−/−^), hepatic steatosis, inflammation, and fibrosis swiftly developed in response to a high fat/high sucrose diet, and these effects were reversed using dietary choline supplementation [86,87,88,89,90]. We [91] and others [86] observed lower hepatic *PEMT* expression in NASH patients relative to NAFLD patients.

Common *PEMT* variants disrupt normal phosphatidylcholine synthesis and have been associated with a heightened predisposition to NAFLD [92,93], which can be further exacerbated by low dietary choline intake [39,94]. The rs7946 variant, which produces a Val-to-Met substitution at residue 175 of the human PEMT protein, has a higher frequency in NAFLD patients compared to those without NAFLD [92,95,96]. The Met isoform of PEMT also exhibits a specific activity that is 40% lower than the wild-type Val isoform [92]. Variant alleles in other common *PEMT* variants, rs1531100 and rs4646365, were associated with a higher risk of liver damage in postmenopausal women under low choline conditions [94].

The effects of choline deficiency on metabolic function may be worsened in postmenopausal women, in part because *PEMT* gene expression is regulated by estrogen [97,98]. As estrogen levels decline, the metabolism and utilization of choline shifts. An RCT to investigate the impact of choline depletion found that postmenopausal women were more susceptible than premenopausal women to developing fatty liver or muscle damage in response to the treatment. Specifically, 80% of postmenopausal women (12 out of 15) developed the conditions, whereas only 44% of premenopausal women (7 out of 16) did [77], suggesting that estrogen levels may mediate variation in choline requirements. A study examining the impact of choline intake on fibrosis severity in individuals with NAFLD from the NASH Clinical Research Network found that postmenopausal women with deficient choline intake experienced significantly worse fibrosis, even after controlling for other factors associated with NAFLD, such as age, race/ethnicity, obesity, elevated triglycerides, diabetes, alcohol use, and steroid use [99]. However, choline intake was not found to contribute to disease severity in children, men, or premenopausal women [99]. Conversely, studies have shown that women with a higher dietary choline intake have a lower risk of developing NAFLD. For instance, in one study, women who reported a higher dietary choline intake had a lower risk of abdominal ultrasound-diagnosed NAFLD [80]. This finding was further supported by another study conducted by Mazidi et al. [100], which found that postmenopausal participants from the National Health and Nutrition Examination Survey (NHANES) in the highest quartile of choline intake had a 26% lower risk of NAFLD compared to those in the lowest quartile.

Research suggests that the effects of estrogen deficiency on choline depletion can be reversed. A study conducted by Fischer et al. [39] found that postmenopausal women who were given exogenous estrogen had a significantly lower risk of developing liver dysfunction in response to a very-low-choline diet, compared to those who received a placebo. While the sample size was small (N = 22), the study suggests that hormone therapy during menopause may help prevent the development of hepatic steatosis in postmenopausal women who have a low dietary choline intake or are unable to produce endogenous choline efficiently due to genetic factors.

Additionally, we observed that postmenopausal women with NASH had significantly lower hepatic *PEMT* expression compared to those with normal liver histology [91]. Our study also found that *PEMT* expression decreased with increasing stage of fibrosis, indicating that postmenopausal women with low estrogen levels may be at a higher risk of disease progression due to reduced *PEMT* expression. This risk would likely be exacerbated by low dietary choline intake [39,77,94,99]. Overall, these findings suggest that MHT and increased choline intake may be potential strategies for reducing the risk of NAFLD in postmenopausal women.

### 3.2. Soy Isoflavones

Soy isoflavones are compounds found in legumes, with a chemical structure, albeit non-steroidal, similar to estrogen. Soybeans and soy products are the richest sources of isoflavones [101], including genistein, daidzein, and glycitein, which are conjugated to different sugars to form glycosides, malonylglucosides, and acetylglucosides [102]. However, the conjugated forms are not biologically active or bioavailable until they are hydrolyzed by intestinal glucosidases to release aglycons [103]. One such aglycon is S-equol, which is a metabolite of daidzein produced by certain intestinal bacteria. Equol has higher antioxidant activity than vitamins C or E; greater biological potency than its precursor, daidzein; and complete bioavailability [104]. Approximately 30% of Western adults and 60% of Asian adults can produce equol after consuming soy foods [105,106]. Individuals who can generate equol may derive the greatest benefit from soy isoflavone consumption [107].

Studies in animals suggest that soy isoflavones can protect against hepatic steatosis and fibrogenesis [108,109], with higher concentrations of soy isoflavones conferring greater protection [110]. Epidemiological studies have found an inverse association between soy food intake and newly diagnosed NAFLD [111]. Dietary intervention trials have further supported the beneficial impact of soy supplementation in NAFLD patients (Table 1) [112,113,114,115,116,117]. Although the human clinical trials conducted to date have varied by study design, treatment protocol, method of liver fat imaging, and outcomes, the results have been largely consistent, indicating a positive effect of soy protein on liver function in NAFLD patients.

Concordant with the consumption of soy isoflavones, the production status of equol may also be an important factor to consider with respect to NAFLD in postmenopausal women. For instance, a study involving 38 NAFLD patients (13 men and 25 women) found that the differences in liver histology by equol production status varied depending on the patient’s sex [108]. Specifically, no differences were observed between male equol producers and nonproducers in terms of histological features. However, in postmenopausal women, the degree of fibrosis and hepatocyte ballooning was significantly higher in equol nonproducers compared to equol producers. Moreover, the percentage of postmenopausal nonproducers with a NAFLD activity score (NAS) ≥ 5 was significantly higher than that of producers, and equol production status was found to be the strongest contributor to the development of NASH in postmenopausal NAFLD patients.

Soy isoflavones may provide unique benefits for women who experience a decline in estrogen levels after menopause due to their estrogen-like effects on metabolism. Studies in postmenopausal women have reported ameliorative effects of soy isoflavones on bone health [119,120,121], menopausal symptoms [120,122,123,124,125], certain types of cancer [120,126,127,128], and obesity [120,129], although not all studies have observed benefits [130,131,132,133,134], and these discrepancies may be due, in part, to differences in the type, dose, or source of isoflavone used. Furthermore, higher levels of adiposity in both peri- and postmenopausal women have been associated with intestinal microflora that cannot metabolize daidzein [135,136]. While it is not yet clear whether the ability to produce equol affects the effectiveness of isoflavones on postmenopausal conditions [104], equol producers have been found to report fewer and less severe menopausal symptoms than non-producers [137]. In contrast, a recent study demonstrated that postmenopausal women consuming a low-fat vegan diet with daily intake of cooked soybeans (86 g) over a period of 12 weeks experienced an 88% reduction in moderate-to-severe hot flashes compared to a control group who did not make any dietary changes, and the degree of improvement in vasomotor symptoms was independent of equol producer status [138].

Currently, there are no studies that have specifically investigated the effects of soy isoflavones on hepatic fat content in postmenopausal women. However, Panneerselvam et al. [139] conducted an animal study using high-fat-diet-fed ovariectomized rats as an experimental model for human menopause. The results showed that when fed a high-fat diet, ovariectomized rats gained weight and developed hepatic steatosis and hypertriglyceridemia. Treatment with soy isoflavone extract improved these conditions. In addition, soy isoflavones reversed the hepatic overexpression of several lipogenic genes that were upregulated by ovariectomy and high-fat diet; and decreased circulating markers of liver injury, including aspartate transaminase, alanine transaminase, lactate dehydrogenase, total protein, and total bilirubin. Although this study was conducted in rats, the results suggest that soy isoflavones may have similarly beneficial effects on liver health and lipid metabolism in postmenopausal women.

The extent of isoflavone intake among postmenopausal women remains poorly characterized. According to the Framingham Heart Study, postmenopausal participants had a median isoflavone intake of 0.15 mg/day (with a range of 0.99–0.24 mg/day), and those in the highest quartile of isoflavone intake had significantly lower plasma triglyceride levels and a lower mean cardiovascular risk factor metabolic score compared to those in the lowest quartile [140]. This is in contrast to Asian countries, where typical soy isoflavone intake ranges from 25–50 mg/day, which is significantly higher than in the United States [141].

While there is some evidence to suggest that soy isoflavones may be beneficial for postmenopausal women, more research is needed to fully understand their effects and potential risks. Furthermore, inconsistent findings in human clinical trials highlight the need for more controlled mechanistic studies in vivo, due to the significant interpersonal variations in the human gut microbiome, which can lead to conflicting outcomes [142]. Soy isoflavone intake may therefore be particularly important for postmenopausal women who are equol producers and do not have gut dysbiosis.

### 3.3. Probiotics

Gut dysbiosis is a condition characterized by an imbalance in the relative abundance of certain bacterial species or groups, which is often accompanied by lower overall diversity of the gut microbiota [143,144]. This condition has been linked to the development and progression of NAFLD [144,145,146]. The imbalance in the gut microbiota can cause increased intestinal permeability, which enables bacterial products such as lipopolysaccharides (LPSs) to enter the circulation, triggering an immune response and leading to systemic inflammation and liver damage. In animal models, LPSs have been shown to promote the development of NAFLD, and high levels of LPSs have been found in the blood of NAFLD patients [147,148]. Moreover, gut dysbiosis can also affect bile acid metabolism, leading to changes in the composition of bile acids produced and excreted into the small intestine. Alterations in bile acid composition can affect lipid absorption and lead to the accumulation of lipids in the liver, thereby contributing to the development of NAFLD [149]. Additionally, dysbiosis can promote the growth of pro-inflammatory bacteria while reducing the abundance of anti-inflammatory bacteria, resulting in chronic low-grade systemic inflammation that may further contribute to NAFLD development [150].

Menopause has been linked to decreased alpha-diversity and changes in the abundance of different bacterial groups [143,151,152,153,154]. Peters et al. [143] conducted a large shotgun metagenomic sequencing study and discovered that postmenopausal women (N = 1027) had a gut microbiome diversity and overall composition that was similar to that found in men (N = 978) and lower than that in premenopausal women (N = 295). Menopause-related changes in the gut microbiome were also associated with an unfavorable cardiometabolic profile. Despite some heterogeneity, several studies of gut microbial composition in postmenopausal women have revealed a lower abundance of *Firmicutes* and *Ruminococcus* and a higher abundance of *Butyricimonas*, *Dorea*, *Prevotella*, *Sutterella*, and *Bacteroides* relative to premenopausal women [155]. However, the implications of these findings are not yet clear as the functions and health effects of these bacteria are not fully understood. Therefore, the health consequences of menopause-related gut microbiome alterations remain to be determined [155].

Several RCTs have investigated the impact of probiotic supplementation on liver-related outcomes in individuals with NAFLD. Despite variations in the types of treatments used, the duration of the interventions, and the outcomes measured across these studies, the findings suggest that probiotic supplementation has beneficial effects on liver health (Table 2). A meta-analysis of 21 RCTs, comprising 1252 participants, identified significant decreases in ALT levels and liver stiffness, and improvement in hepatic steatosis in response to probiotic/synbiotic use [156]. An independent meta-analysis of 18 studies demonstrated that the use of probiotics as adjuvant therapy for NAFLD patients improved liver function and reduced levels of liver transaminases, such as ALT, AST, and GGT, particularly when the duration of treatment was greater than 12 weeks [157]. This analysis also indicated positive effects on various cardiometabolic measures, including levels of triglycerides, total cholesterol, fasting blood glucose, insulin, insulin resistance, and BMI associated with probiotic supplementation. Based on this evidence, therapies aimed at targeting the gut microbiome could be a promising approach for the management of NAFLD.

To date, there have been no studies specifically examining the effects of probiotic supplementation on liver health in postmenopausal women. Nevertheless, a RCT in 81 postmenopausal women with obesity found that a 12-week course of multispecies probiotic supplements led to reduced levels of visceral and subcutaneous fat, lower waist circumference, and improved cardiometabolic markers, including uric acid, glucose, insulin, and HOMA-IR [167], all of which would be expected to yield hepatic benefit. However, 24 weeks of probiotic supplementation in individuals with biopsy-proven NASH (15 female/8 male; mean age 51.7 ± 11.4 years) did not result in any significant changes in liver function (ALT, AST, or GGT) or metabolic health (i.e., fasting glucose, HbA1c, insulin, triglycerides, cholesterol) [168]. Although menopause was not specifically considered in this study, the age range of the female participants is consistent with the average age of menopause in the United States [169]. The discrepancy between studies may be due to the experimental design and differences in both the study sample size and clinical endpoints. In general, the positive results of previous RCTs conducted in male and female participants of various ages [170] suggest that probiotic supplementation may also be a useful adjuvant therapy for postmenopausal NAFLD patients with gut dysbiosis.

Overall, menopause appears to be associated with lower gut microbiome diversity and a shift toward greater similarity to the male gut microbiome. However, further studies for identifying consistent and reproducible changes that occur in the taxa as a result of menopause are warranted, as are studies to better understand the contribution of the gut microbiota to menopause-related NAFLD risk and the impact of menopausal hormone therapy on the gut microbiome-NAFLD axis.

## 4. Conclusions

**Figure 2:**
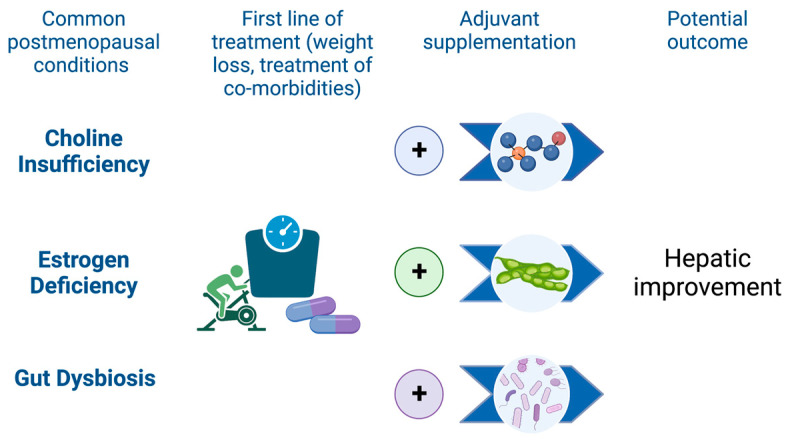
Adjuvant nutritional strategies may enhance primary NAFLD management in postmenopausal women. Weight loss, lifestyle factors, and clinical management of comorbidities remain the cornerstones of therapy for NAFLD. Nutritional factors may further enhance the effectiveness of first-line therapy in specific subsets of postmenopausal patients. Increased choline intake, either through choline-rich foods or supplements, may help to resolve liver function in postmenopausal women who have a choline-depleted diet, genetic risk factors, or estrogen deficiency. Consumption of soy foods may provide hepatic benefit to postmenopausal women, especially those who can produce equol, through estrogen-like effects. In postmenopausal women with gut dysbiosis, supplementation with probiotics or consumption of fermented foods may correct microbial imbalance, leading to improvements in hepatic function. (Figure created with BioRender.com, accessed on 31 May 2023).

Following menopause, women experience a higher risk of NAFLD, as well as higher rates of advanced hepatic fibrosis and greater mortality [10]. Managing weight, increasing physical activity, and controlling comorbidities are essential strategies to reduce the burden of NAFLD in this population. In addition, specific subsets of postmenopausal women, such as those who experience chronic choline deficiency, estrogen deficiency, or gut dysbiosis, may benefit from targeted nutritional interventions that include consumption or supplementation with choline, soy isoflavones, or probiotics (Figure 2). There is promising evidence supporting the potential benefits of these nutritional factors for NAFLD prevention and treatment, and further research is warranted to confirm their effectiveness in alleviating hepatic steatosis in postmenopausal women. In the meantime, it remains crucial for women to follow a healthy diet that is rich in whole foods, low in processed foods, and balanced in nutrients to reduce the risk of NAFLD following the menopausal transition.

## Figures and Tables

**Table 1:** Effects of soy product consumption on liver related outcomes in adults with NAFLD.

Country	N	Intervention	Duration * (wk)	Key Liver Outcomes	Ref.
Iran	45	30 g soy nut	8	Reduced ALT and AST levels	[116]
Iran	70	240 mL soy milk	8	Reduced ALT, no change in fatty liver grade or AST, GGT, ALP levels	[112]
Germany	22	Soy meal replacement (83% soy protein isolate)	24	Reduced ALT and liver fat, but not different from the lifestyle modification control group	[118]

* Duration is shown in weeks. Abbreviations: ALP: alkaline phosphatase; ALT: alanine transaminase; AST: aspartate transaminase; GGT: gamma-glutamyltransferase.

**Table 2:** Effects of probiotic supplementation on liver-related outcomes in adults with NAFLD.

Country	N	Intervention	Duration *	Key Liver Outcomes	Ref.
Spain	28	*Lactobacillus bulgaricus* and *Streptococcus* *thermophilus*	12	Reduced ALT, AST, and GGT levels	[158]
Iran	72	Probiotic-enriched yogurt	8	Reduced ALT and AST levels	[159]
Egypt	30	*Lactobacillus acidophilus*	4	Reduced ALT and AST levels, no change in AUS findings	[118]
Ukraine	75	*Lactobacilli*, *Bifidobacteria*, and *Streptococcus thermophilus*	12	Decreased ALT levels and liver stiffness	[160]
Ukraine	58	Multistrain probiotic	8	Decreased fatty liver index, reduced AST and GGT levels	[161]
Korea	68	Multistrain probiotic	12	Decreased intrahepatic fat	[162]
Iran	89	Multistrain probiotic	12	Decreased ALT, AST, GGT, and ALP levels	[163]
Iran	53	*Bacillus coagulans* plus inulin	12	Decreased levels ALT and GGT levels, improved steatosis	[164]
Malaysia	33	Multistrain probiotic	24	No significant changes in steatosis, inflammation, fibrosis, or ALT levels	[165]
United Kingdom	35	VSL#3	10	No significant changes in transaminases, fibrosis risk score and ASQ	[166]

* Duration is shown in weeks. Abbreviations: ALP: alkaline phosphatase; ALT: alanine transaminase; ASQ: acoustic structure quantification; AUS: abdominal ultrasound; AST: aspartate transaminase; GGT: gamma-glutamyltransferase.

## Data Availability

Not applicable.
